# Multidisciplinary Software Design for the Routine Monitoring and Assessment of Pain in Palliative Care Services: The Development of PainCheck

**DOI:** 10.1200/CCI.18.00120

**Published:** 2019-10-02

**Authors:** Matthew J. Allsop, Owen Johnson, Sally Taylor, Julia Hackett, Peter Allen, Michael I. Bennett, Bridgette M. Bewick

**Affiliations:** ^1^ University of Leeds, Leeds, United Kingdom; ^2^ X-Lab, Leeds, United Kingdom

## Abstract

**PURPOSE:**

The use of health information technology (HIT) to support patient and health professional communication is emerging as a core component of modern cancer care. Approaches to HIT development for cancer care are often underreported, despite their implementation in complex, multidisciplinary environments, typically supporting patients with multifaceted needs. We describe the development and evaluation of an e-health tool for pain management in patients with advanced cancer, arising from collaboration between health researchers and a commercial software development company.

**METHODS:**

We adopted a research-led development process, involving patients with advanced cancer and their health professionals, focusing on use within real clinical settings. A software development approach (disciplined agile delivery) was combined with health science research methods (ie, diary studies, face-to-face interviews, questionnaires, prototyping, think aloud, process reviews, and pilots). Three software iterations were managed through three disciplined agile delivery phases to develop PainCheck and prepare it for use in a clinical trial.

**RESULTS:**

Findings from development phases (inception, elaboration, and construction) informed the design and implementation of PainCheck. During the transition phase, where PainCheck was evaluated in a randomized clinical trial, there was variation in the extent of engagement by patients and health professionals. Prior personal experience and confidence with HIT led to a gatekeeping effect among health professionals, who were reluctant to introduce PainCheck to patients. Patients who did use PainCheck seemed to benefit, and no usability issues were reported.

**CONCLUSION:**

Health science research methods seemed to help in the development of PainCheck, although a more rigorous application of implementation science methodologies might help to elucidate further the barriers and facilitators to adoption and inform an evidence-based plan for future implementation.

## INTRODUCTION

For patients with cancer, research shows that pain is frequent, burdensome, and undertreated.^1-4^ More than two thirds of patients with cancer will experience pain during the advanced, metastatic, or terminal stage of their cancer.^4^ Pain is a major source of suffering for these patients, having adverse effects on their quality of life, leading to unplanned hospital admissions with uncontrolled symptoms,^5^ and negatively affecting caregivers.^6^ Although a number of evidence-based clinical practice guidelines are available, pain continues to be undertreated.^7,8^ Barriers to effective pain management have been identified at the patient (eg, reluctance to complain about symptoms, fear of pain), health professional (eg, inadequate assessment of pain, reluctance to prescribe or monitor analgesics), and health care system levels (eg, ineffective communication about data on pain, preventing patient access to timely analgesia).^9^

Information and communication technology, and specifically health information technology (HIT), can support patient and health professional communication as part of cancer care^10^ and facilitate approaches that target known barriers to pain management. Examples include HIT use to capture patient-reported outcomes,^11-13^ self-reported symptom information,^14^ and delivery of educational interventions.^15^ Well-validated patient-reported outcomes have been developed specifically for the oncology setting (eg, the Patient-Reported Outcomes version of the Common Terminology Criteria for Adverse Events^16^). Efforts to leverage HIT to capture and use such patient-reported outcomes have been reported.^17,18^ When HIT is used in such ways, it can have a positive impact on care, reducing symptom distress,^15^ improving quality of care,^12^ and enabling real-time reporting to support earlier clinical decision making.^19^ For the management of cancer pain, technology can be used as an intermediary for patients to report their pain,^20^ addressing known barriers to good pain management. HIT, used in this way, has both patients and providers as end users, augmenting communication beyond face-to-face consultation. However, HIT systems for use in advanced cancer are at an early stage of adoption, with little information on how HIT tools are being designed and developed, leading to a lack of clarity on the best methods for development.^21^

**CONTEXT**

**Key Objective**
How can information and communication technology (ICT) systems be developed and implemented rigorously in the context of cancer and palliative care services?
**Knowledge Generated**
Multidisciplinary teams are able to work and communicate effectively to undertake user involvement and generate valuable and rich data that can meaningfully inform software design decisions for cancer and palliative care services. Subsequent implementation of ICT systems in palliative care must ensure that health professionals are well trained, are supported in ICT use, and perceive benefits for patients; otherwise, uptake and engagement could be adversely affected.
**Relevance**
Our approach, detailing methods for engaging patients receiving palliative care and their health professionals from conception to implementation, provides a framework to guide rigorous development of future e-health systems intended for use in cancer and palliative care services.

HIT systems are typically complex interventions. When developed in the context of care for patients with advanced cancer, system implementation often occurs within challenging, complex, multidisciplinary environments. Patients with advanced cancer are often supported by palliative care services in acute, community, and hospice settings.^22^ Palliative care services support people with progressive, life-threatening diseases with no possibility of obtaining remission or stabilization or modifying the course of the illness, often with accompanying symptoms that may require pain management.^23^ The complexity of palliative care delivery models for patients with often complex needs highlights the importance of developing HIT systems that are informed by and aligned with the needs of end users.^24^

Approaches to software development have a long history of gathering the needs of users through developing a list of their requirements based on needs and preferences.^25^ Modern software development teams are typically organized into small groups that work flexibly and collaboratively with a range of stakeholders to inform the development of an HIT system or product. The identification of user requirements as part of this process can lead to the development of HIT systems that are more successful in supporting patients with complex needs and symptoms.^26-28^ Currently there is a lack of literature to guide method selection to support HIT systems for pain management in cancer care.^21^ This report describes our experience of combining modern software development with health science research methods to create PainCheck, an HIT system designed to overcome known barriers to effective pain management for patients with advanced cancer. PainCheck was specifically developed to be suitable for a clinical trial as part of a complex intervention. It has now been implemented in palliative care settings.^29^ We document the methodology adopted for undertaking research and working with system developers, alongside reporting the experience of patient and health professional users of PainCheck in the context of routine care as part of a clinical trial. Our aim is to share our methodology to provide a template to support research-led development of HIT systems for palliative care.

## METHODS

### Context of HIT System Development

PainCheck stemmed from a large research program (IMPACCT [Improving the Management of Pain From Advanced Cancer in the Community; ISRCTN registry No. 18281271]) in the United Kingdom,^29^ with a specific work stream dedicated to routine assessment and monitoring of pain in patients with advanced cancer. Complementary parallel work streams explored pathways of care for patients with advanced cancer, the role of educational interventions to support self-management of pain, opioid-prescribing practices, and the cost effectiveness of reducing pain and related distress. A multidisciplinary team was formed to develop PainCheck. The team was led by a psychologist and included social scientists, palliative care professionals, public and patient involvement representatives, and a private software company (X-Lab, Leeds, United Kingdom). X-Lab was contracted a set amount of funding to perform the development work. X-Lab had previously developed QTool, an electronic online questionnaire management software suite. QTool is used by health care practitioners and researchers to build and schedule complex questionnaires that can be completed by patients and clinical staff. Examples of its use include patient-reported outcomes in cancer survivors^30^ and self-report and management of adverse events during cancer treatment.^31^ QTool was selected as a starting point for the development of PainCheck.

### Overview of Approach to HIT System Development

The software development team consisted of three developers and a business analyst, all trained in agile methods.^25^ Development followed the disciplined agile delivery (DAD) methodology, which is a formal structure used by software developers to guide HIT system development from the initiation of ideas through implementation and eventual retirement.^32^ The DAD methodology shares principles of approaches often used to develop interventions in health research, such as user-centered design^33^ and participatory design,^34^ where the stakeholder, or end user of a technology or product, is central to its design and development. Working within the DAD framework provided a clear development process for the system developers. It also provided clear time points for the research team, highlighting when findings from research activities were required by system developers to inform the next stage of development. The research team adopted a mixed-methods approach, combining surveys with qualitative interview studies and usability testing.

The DAD framework plans system development over four phases: inception, elaboration, construction, and transition. The inception phase of the project began with the team generating a working technical specification document, which outlined the planned components and functions that were initially deemed necessary for an HIT system for pain management (eg, ability for reporting of pain scores, communication between patient and health professional). During the inception phase and subsequent elaboration and construction phases, we conducted a range of research activities with patients, their caregivers, and health professionals to guide the subsequent development of the HIT system. Throughout each phase of development, the following process was followed:

1. The research team synthesized findings from its research activities for the software development team;
2. The research findings were used by software developers to update and modify the technical specification document for the HIT system; and
3. The revised technical specification document was used to update the HIT system and provide a prototype matching the revised technical specification document.

The research team used the most recent prototype during research activities with patients, caregivers, and health professionals.

### Procedure for HIT System Development

Before involvement of patients with advanced cancer, caregivers, and health professionals, two preliminary activities were undertaken as part of the inception stage:

1. Assessing the quality and completeness of data captured by the QTool infrastructure; and
2. Engaging with a member of our patient and public involvement group to undertake preliminary exploration of the context and experience of patients with advanced cancer and their caregivers, alongside reviewing study documentation (Data Supplement provides details and examples of involvement).

The quality and completeness of data captured through QTool were tested using a population of people with chronic pain,^35^ assessing the quality of data collected and stored by QTool.

**FIG 1.**
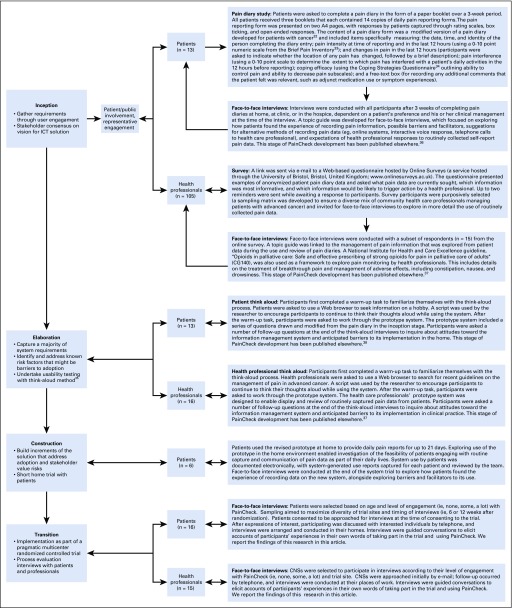
Overview of the methods used during the inception, elaboration, construction, and transition phases of PainCheck development. CNS, clinical nurse specialist; ICT, information and communication technology.

After these preliminary activities, user engagement was structured within the four phases of DAD methodology: inception, elaboration, construction, and transition. Figure 1 outlines the different stages of development; methods applied at each stage, including participant numbers; and citations for research activities across the inception, elaboration, and construction phases that have been published previously.^21,35-38^ At the end of each phase, research activities were summarized by the health researchers and outlined in a spreadsheet, with actions for the research team and proposed software development changes that aligned with the needs and preferences of patients, caregivers, and health professionals. Software requirements were documented and discussed with the software developers to determine how these translated into appropriate adaptations to QTool. Software developers then used a final list of requirements to develop another iteration of the HIT system using QTool.

The final system, called PainCheck, was evaluated as part of a pragmatic multicenter randomized controlled trial. A full protocol for the trial has been published.^29^ Patients were recruited from six of the eight participating oncology clinics across the United Kingdom who met the eligibility criteria (outlined in the transition section of Table 1). A process evaluation was undertaken during this stage as part of the trial. This involved semistructured interviews being conducted at 6 or 12 weeks postrandomization with patients with advanced cancer and community palliative care (CPC) nurses (sampling approaches are outlined in the Data Supplement). Interviews sought to gather perspectives on the implementation of PainCheck to support pain management for patients with advanced cancer in the context of routine palliative care. Data collection and analysis were undertaken by the research team. Additional details of the approach to analysis are outlined in the published trial protocol.^29^

**TABLE 1.**
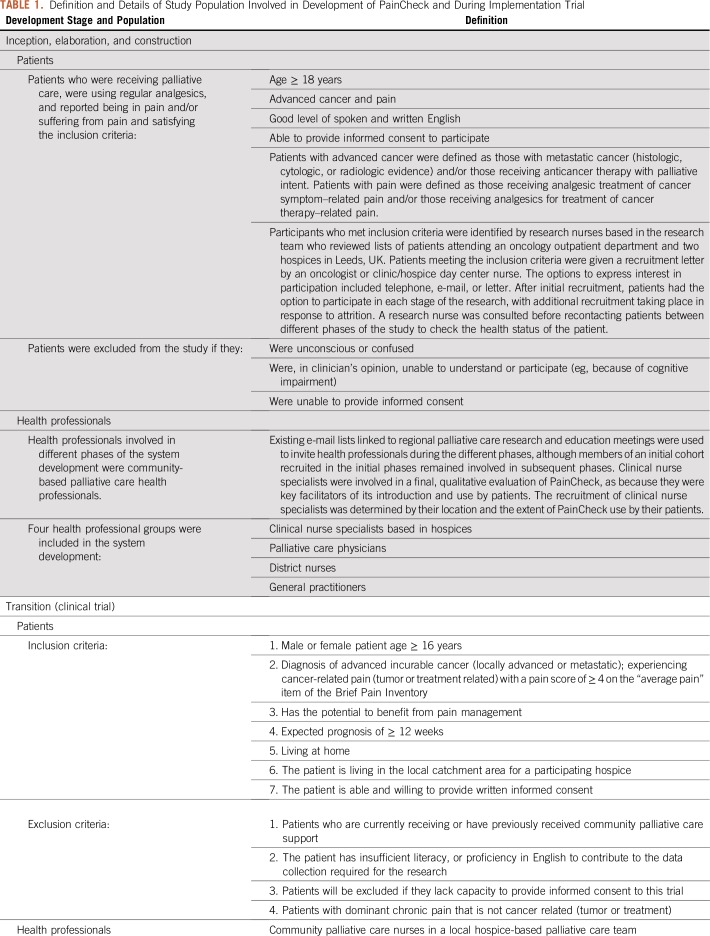
Definition and Details of Study Population Involved in Development of PainCheck and During Implementation Trial

### Human Investigations

The investigators performed the human investigations after approval by a local human investigations committee (National Research Ethics Service Committee Yorkshire and the Humber–South Yorkshire; 13/YH/0054). They obtained informed consent from each participant. The name of the woman with cancer outlined in the Data Supplement, Barbara, was not changed, because Barbara was aware of the potential wider use of the data generated by Peter Allen, the husband and caregiver of Barbara and coauthor of this report, who agreed to its publication. This position was discussed and agreed with the local institutional ethics board of the Faculty of Medicine and Health at the University of Leeds (Leeds, United Kingdom).

## RESULTS

### Findings From the Inception, Elaboration, and Construction Phases

We present the findings from the inception, elaboration, and construction phases in Table 2. These outline the user requirements that were extracted from research activities undertaken at each stage of development. Although the research methods and findings have been published elsewhere, the user requirements extracted from this work have not been reported previously. For each phase, Table 2 lists the evidence generated and subsequent action by the research team and software developers.

**TABLE 2.**
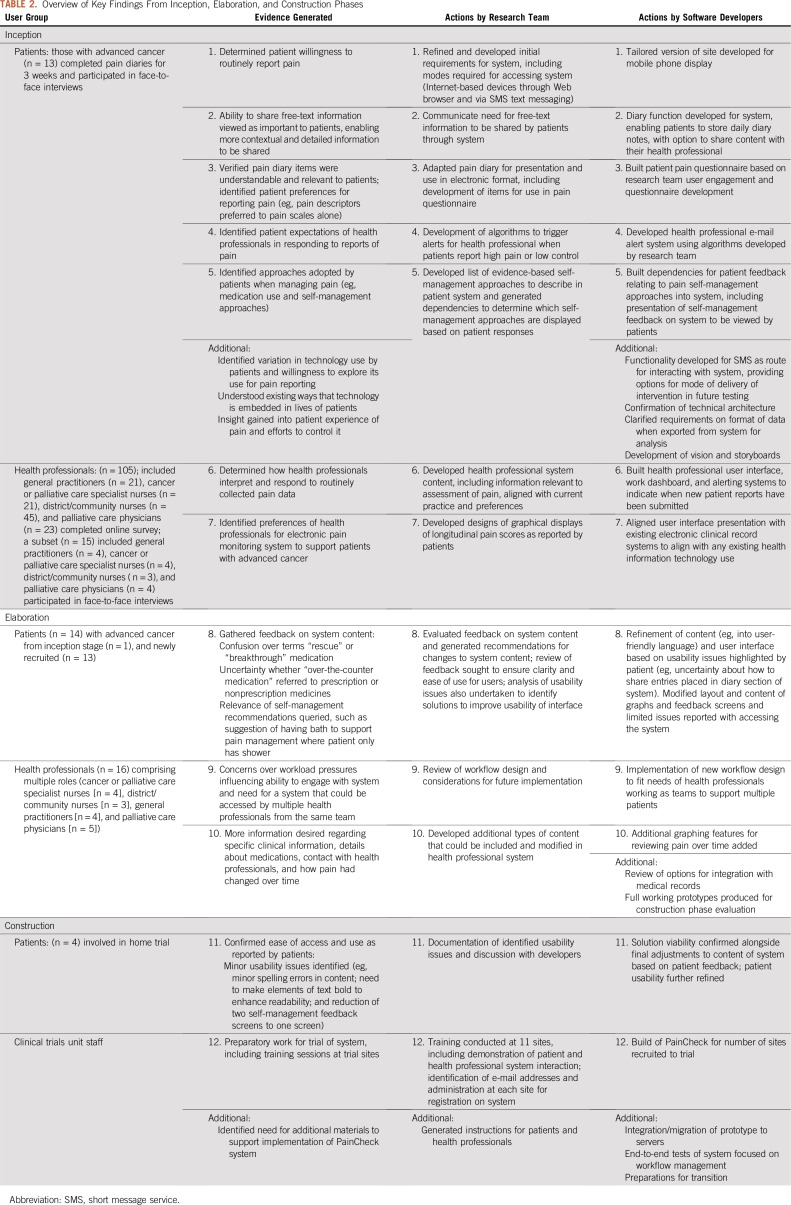
Overview of Key Findings From Inception, Elaboration, and Construction Phases

### Findings From the Transition Phase

**FIG 2.**
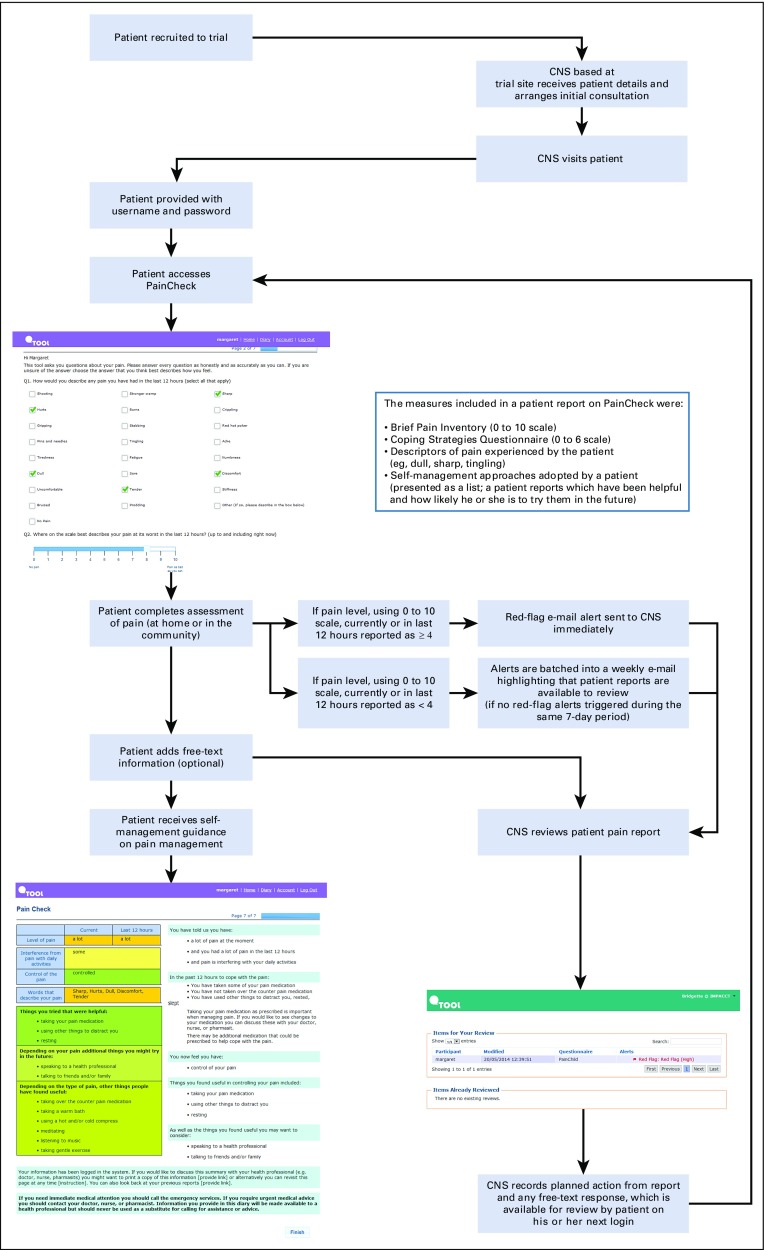
Schematic of PainCheck system implementation in the context of a clinical trial, with screenshots. CNS, clinical nurse specialist.

The design and content of PainCheck were finalized before its inclusion in a pragmatic multicenter randomized controlled trial. The way in which PainCheck was introduced and used in the context of the trial is outlined in Figure 2, alongside examples of system content provided for both patients and health professionals. Full details of the intervention content have been published.^29^

**FIG 3.**
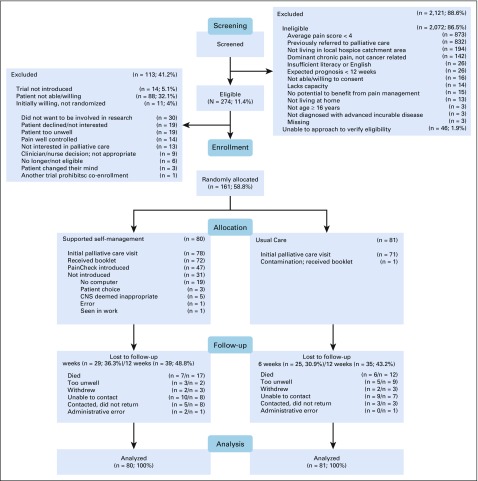
CONSORT diagram of participant progress through the phases of the trial and numbers of patients who engaged with the PainCheck intervention. CNS, clinical nurse specialist.

In total, 47 of the 80 intervention participants were introduced to PainCheck. The key findings from the process evaluation interviews undertaken as part of the clinical trial are listed in Table 3. As shown in Figure 3, not having a computer was the most common reason for patients not to use PainCheck. Patient access to PainCheck was also influenced by health professionals, and CPC nurses had the role of facilitating and monitoring patient interaction with PainCheck. Some patients were not introduced to PainCheck to avoid what CPC nurses perceived as an unnecessary additional burden for them. For patients, a lack of familiarity with HIT or not having an Internet connection at home also influenced the perceived value and uptake of PainCheck.

**TABLE 3.**
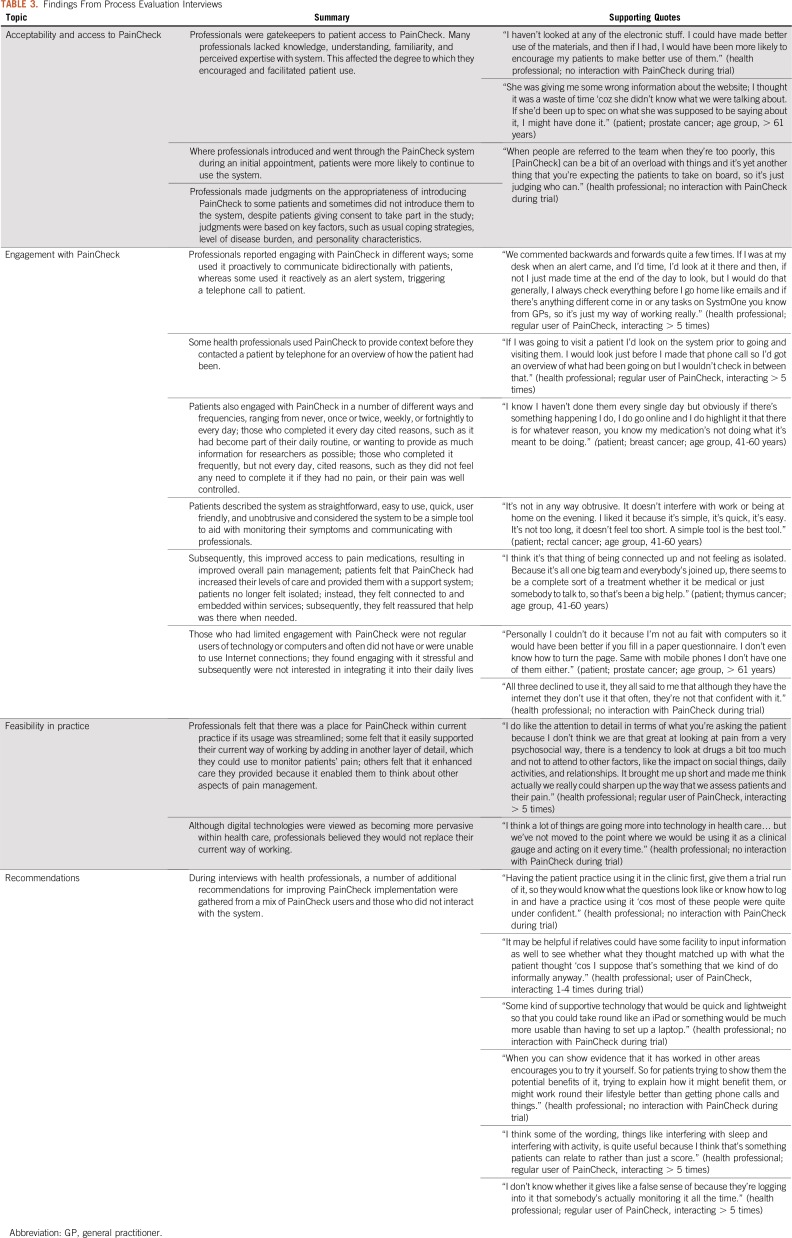
Findings From Process Evaluation Interviews

A more detailed overview of the number of patients recruited to the trial, alongside the numbers of patients who engaged with the PainCheck intervention, is provided in Figure 3. Of those introduced, varying levels of engagement were identified. Across patient participants, there were those who completed no reports during the trial (n = 15), alongside those completing reports one to two times (n = 9), three to four times (n = 6), five to nine times (n = 7), 10 to 19 times (n = 5), and more than 20 times (n = 5). For those patients who completed reports, a large proportion (n = 27; 84%) used the diary function, opting to send free-text reports to their health professionals. Where patients and CPC nurses did interact through PainCheck, a range of approaches was identified. There was a mix of proactive and reactive styles of interaction by CPC nurses, accompanied by varied frequencies in the timing and extent of PainCheck use by patients. Proactive use of PainCheck involved CPC nurses reviewing patient reports to plan and manage their workload, alongside sending messages directly to patients. Reactive styles involved CPC nurses being prompted to review and interact with PainCheck when alerted by submission of reports suggesting high levels of pain were being experienced by a patient. Despite variation in use, both patients and CPC nurses who engaged with PainCheck reported benefits to overall pain management. CPC nurses saw systems like PainCheck as having a place in current practice, but they were clear that the role of PainCheck should be to enhance existing care delivery rather than replace it.

## DISCUSSION

This article reports the development of an HIT system for palliative cancer care across all stages of development; to our knowledge, this has not previously been reported in systems supporting patients with advanced cancer.^21^ The HIT system, PainCheck, was developed collaboratively by researchers and software developers across four phases of development. This approach combined modern system development with methodic approaches by health researchers, enabling a feasible and reproducible approach to HIT development. Involvement of patients and health professionals during each phase ensured that a focus on user needs and preferences informed the design process and that numerous problematic aspects of the system were identified and rectified. This was achieved in the context of palliative care delivery, which involved multiprofessional teams and patients with advanced disease, some of whom were close to death. The documentation of our approach and the experience of PainCheck users are intended to inform future research-led development of HIT systems for palliative care. The absence of usability issues identified with PainCheck may have arisen through continuous user involvement during HIT system development.^40^

In the context of the trial, barriers to uptake of PainCheck were identified. For patients, their own familiarity with technology, alongside access to a computer and the Internet, was a barrier. For health professionals supporting the introduction and use of PainCheck in the community, barriers included a lack of confidence and familiarity with PainCheck, and HIT generally, which influenced decision making around whether they introduced the system to patients. This may have been combined with a common focus by health professionals on the vulnerability of patients, coupled with an emphasis on the duty to protect patients, when considering suitability for research.^41^ Reluctance to introduce PainCheck may have also been influenced by the protocol for delivery of health professional training on using the system. Training occurred during site setup for the trial, often occurring months before recruitment of the first trial participant. This may have led to health professionals being less confident in the use of PainCheck. Enhancing delivery of training to ensure it occurs close to planned system use may reduce the likelihood of such gatekeeping during future implementation of PainCheck. It may also be important to emphasize the intended value and benefits of an HIT system for patients to address health professional uncertainty and concerns around its impact on patients.

Patients who engaged with PainCheck did report benefits (eg, feeling more connected with their care team, perceived improvements in pain management), but there was wide variation of interaction with the system. This highlighted the need to consider both the technology and behavioral aspects surrounding PainCheck. Use alone does not provide a valid indicator of engagement.^42^ Future development will need to consider the wider context and mechanisms of action surrounding PainCheck to understand how best to measure and target improvements in engagement. This will require consideration of the complexity of the pain experience and its meaning for patients with advanced cancer.^37^ Another consideration is the need to explore ways of augmenting PainCheck for patients who do not use a computer or are not familiar with HIT (ie, one quarter of trial participants in the intervention arm of the trial involving PainCheck). The rationale for developing PainCheck was to increase routine monitoring and assessment of pain using an HIT system. Future iterations of PainCheck could also explore approaches such as voice response technology to gather data by telephone, an approach that has been implemented previously for symptom management in palliative care populations.^43^

The development of PainCheck highlighted a tension between the continuous, iterative development of HIT systems by software developers and the controlled processes of formal evaluation in research. Approaches to evaluation that incorporate, for example, randomized controlled trials are only recommended when the intervention and its delivery package are stable. These can be implemented with high fidelity, and there is a reasonable likelihood that the overall benefits will be clinically meaningful (ie, improved outcomes or equivalent outcomes at less cost).^44^ Within current clinical trial design, there is not sufficient scope for ongoing, iterative development of HIT-based interventions. This issue requires attention to ensure that the development and evaluation of e-health tools for cancer care keep pace with efforts to increase the use of ever-evolving HIT systems. Rightly, in this context, the demands for rigorous evidence underpinning HIT are increasing. For example, the UK Medicines and Healthcare Products Regulatory Agency classifies some software as a medical device,^45^ requiring high standards of quality certification and evaluation, extending from CE marking to more formal regulation. However, although prospective exploration of user perspectives and forecasting of issues are essential during system development, these activities may identify the need for a system to be modified. The development of more nuanced experimental approaches that enable evaluation alongside ongoing and continuous adaptation of systems could facilitate simultaneous development and rigorous evaluation of HIT systems. This challenge echoes literature on the development of quality improvement interventions, with the need to reconcile pragmatism (eg, the generation of HIT systems by software developers) and research rigor (eg, understanding the underlying mechanisms of HIT interventions and the influence of contextual factors).^46^ Solutions may arise in the development of trial methodology aimed at minimizing the risks of in-trial changes to intervention technologies and maximizing the potential for knowledge acquisition.^47^

This research has limitations. It was undertaken in the context of a research program with a preplanned schedule for system development. This reflects a common approach required for academic research, where methodology is often determined and fixed before obtaining funding. In this study, we had specific points for liaising with developers, and these were constrained by a predetermined budget, limiting the extent to which desired system features might be included. Furthermore, the design of the trial in which PainCheck was implemented may have inadvertently reduced uptake of the system by patients through, for example, the timing of health professional training. The resultant low uptake by patients limited our ability to fully understand factors that influenced interaction and use of PainCheck. Future evaluation of PainCheck could benefit from an alternative trial design, such as a stepped-wedge cluster design,^48^ where sequential introduction of an intervention across sites may avoid long delays between site recruitment and introduction of PainCheck to trial participants.

In conclusion, the use of HIT systems to support patients with advanced cancer is a key area for improving health care and is at an early stage of development. Developing reliable, scalable HIT systems, sharing best practices, and ensuring transparency throughout system development are crucial. Although HIT and care coordination for individuals with complex needs are high priorities for quality improvement in health care, empirical guidance on its development and implementation is lacking.^49^ The use of an overarching framework, borrowed from software development methodology, provided a reproducible structure to interaction and information sharing across our team. The multidisciplinary approach adopted in this research enabled cooperation between health researchers and software engineers, a crucial component in e-health design,^50^ creating an intervention for a palliative cancer care clinical trial.
